# Association between Psychological Flexibility and Health Beliefs in the Uptake of Influenza Vaccination among People with Chronic Respiratory Diseases in Hong Kong

**DOI:** 10.3390/ijerph13020155

**Published:** 2016-01-23

**Authors:** Kin Wai Cheung, Yim Wah Mak

**Affiliations:** 1 Ruttonjee Hospital, 266 Queen’s Road East, Wan Chai, Hong Kong, SAR, China; ckw106@ha.org.hk; 2 School of Nursing, The Hong Kong Polytechnic University, Hung Hom, Kowloon, Hong Kong, SAR, China

**Keywords:** chronic respiratory diseases, health beliefs, influenza vaccination, psychological flexibility

## Abstract

It is common for elderly people and those with such chronic disorders as respiratory diseases to suffer severe complications from influenza, a viral infection. The voluntary uptake of vaccination is vital to the effectiveness of influenza prevention efforts. The Health Belief Model (HBM) is the most commonly used framework in the field of vaccination behavior to explain the decision that people make to accept or refuse vaccination. In addition, psychological flexibility is considered helpful in causing people to be open to adopting new practices that are consistent with their values. This study examined the role of psychological flexibility and health beliefs in predicting the uptake of influenza vaccination among people in Hong Kong. Eligible participants were Hong Kong permanent residents aged 18 years or above with a history of chronic respiratory diseases (CRD). A convenience sample of 255 patients was recruited to participate in a cross-sectional survey in which HBM components and factors of psychological flexibility were assessed. The following variables were found to be significant predictors of vaccination: age, smoking status, comorbidity, previous hospitalization, perceived susceptibility, perceived severity, and psychological flexibility. Enhancing psychological flexibility might be a potential new direction for motivating people to accept influenza vaccination.

## 1. Introduction

Chronic respiratory diseases (CRD) are defined as chronic diseases of the airways and other structures of the lungs, and include asthma, respiratory allergies, chronic obstructive pulmonary disease (COPD), occupational lung diseases, sleep apnea syndrome, and pulmonary hypertension [1]. They constitute a serious public health burden throughout the world, and affect hundreds of millions of people of all ages, particular elderly people. CRD have a significant impact on the quality of life of affected individuals, and can lead to disability and premature death [2]. The World Health Organization (WHO) has predicted that CRD will be the third leading cause of death globally by 2030 [1]. In Hong Kong, CRD are the fifth leading cause of death [3]. Studies have documented that approximately 25% of the population aged 60 or above suffer from CRD [4], with about 8% of patients being admitted to public hospitals because of COPD [5].

The majority of cases of morbidity and mortality associated with CRD are due to acute exacerbations [6]. These may be caused by a number of factors, such as air pollution, the withdrawal of medications, or changes in temperature, but the most common cause is a viral infection (30%–60%) of the trachea-bronchial tree [6,7,8]. National and international respiratory societies have recommended annual influenza vaccinations as a key strategy for the optimal management of CRD [9,10,11,12]. An annual vaccination can reduce rates of mortality and serious lower respiratory infections requiring hospitalization in people with CRD [13]. A recent study investigating the effectiveness of an influenza vaccine in preventing hospitalization due to influenza found that the vaccination reduced influenza-related hospitalizations among people with CRD from 64% to 35% [14]. The voluntary uptake of the vaccination is vital to the effectiveness of influenza prevention efforts. The finding of low uptake rates (19%–58.5%) in various populations has invigorated efforts to better understand the reasons for the uptake or refusal of the influenza vaccine [15,16,17,18]. Understanding the factors behind the vaccination behavior of people with CRD will be crucial for developing effective vaccine promotion activities, and empower people with CRD to make a rational decision on whether to take the influenza vaccine.

There may be multiple factors contributing to the acceptance or refusal of vaccination, some known and some unknown. In this regard, an extensive review of the literature was carried out to identify the existing evidence on vaccination behavior. Previous studies have reported that people who are older [19,20,21,22], married [23,24,25,26], better educated [27,28], non-smokers [22,25,26], have chronic illnesses [16], no limitations on their activities [29], or who have medical insurance [29] are more likely to receive the vaccine than those without these characteristics. Conversely, research has shown that people who are allergic to medication [16] or smokers [30] are less likely to be vaccinated. The findings on the relationship between gender and the uptake of the influenza vaccine have been inconsistent. Some studies found a significant gender difference in the uptake of the influenza vaccine, with women more likely to be vaccinated [23,29], while other studies found the male participants more inclined to receive the vaccine [30].

In summary, all of the reviewed studies had been conducted in Western countries, and most of them had been carried out in community settings. The variables in these studies had been covered in previous studies, and included socio-demographics, lifestyle, co-morbidities, the utilization of health services, medication use, and health beliefs. Almost all of the factors had been generated by reviewing previous research on seasonal influenza vaccinations and expert consultations. Research on influenza vaccinations has primarily focused on high-risk populations, such as pregnant women and healthcare workers. Only a few recent isolated efforts have addressed the vaccination behavior of people with CRD. A scant few studies have been published on influenza vaccinations among Chinese populations. It should be noted that vaccination rates for people with CRD vary greatly, from 19.9% to 71% [16,22,23,24,25,28], still far behind the 2020 target to increase the percentage of high-risk adults to the rate of 90% that is common in developed countries [31].

Moreover, a number of conceptual frameworks have been developed in an attempt to explain why individuals choose to be vaccinated against influenza. The most commonly used framework is the Health Belief Model (HBM) [32], which emphasizes attitudes, beliefs, and knowledge of vaccination as the primary determinants of vaccine uptake in a variety of populations [5,20,21]. Studies have found that those who did not receive the influenza vaccine frequently cited the perceptions that they are not susceptible to influenza and that influenza is a mild disease as their reasons for not getting vaccinated [22,23]. In Hong Kong, health education on influenza vaccination has been incorporated into pulmonary rehabilitation programs. Although many health professionals have used the HBM to obtain information to guide the development of strategies to foster influenza vaccination [33,34], vaccine uptake rates remain suboptimal. This finding illustrates a significant issue: In determining how to come up with an intervention to encourage individuals to get vaccinated against influenza, it may not be adequate to simply examine the health beliefs of individuals. Alternate or additional explanations must be considered to capture some of the more sensitive nuances of the decision to receive an influenza vaccination.

An increasing number of recent studies support the view that there is a relationship between the refusal to be vaccinated and various fears and anxieties. It is possible that such emotions as the fear of catching influenza or concerns about the safety and efficacy of the influenza vaccine play a role in determining whether or not a person decides to get vaccinated. Some researchers have linked the refusal to be vaccinated with trait anxiety, such as concerns about the side effects of the flu vaccination [35,36]. Some patients have also refused to receive vaccinations for fear of the needle [37]. Conversely, a recent study reported a relationship between individuals’ readiness for change and a higher prevalence of vaccination [38]. These studies provide initial support for the view that an acceptance-based style of regulating the emotions promotes vaccination uptake, while an avoidance-based style inhibits action.

Psychological flexibility (PF) is defined as “the ability to contact the present moment more fully as a conscious human being, and to change or persist in behavior when doing so serves valued ends” [39]. By contrast, experiential avoidance (EA) is defined as an unwillingness to remain in contact with certain private experiences, such as thoughts, emotions, and physical sensations, accompanied by counterproductive or harmful attempts to alter or avoid these experiences. EA is a pathological process that underlies many forms of psychopathology [39]. PF and EA are in fact the same concept on a continuum. In the short run, people trying to avoid or escape negative private experiences such as anxiety and self-doubt often subsequently increase the frequency with which they engage in these unwanted experiences [30,39,40]. EA, one of the core concepts in psychological inflexibility, is particularly prevalent in patients with chronic health problems, who often increase the use of avoidance behaviors to control or eliminate unwanted private experiences such as anxiety, worry, and fear [39]. An understanding of the influence of PF would be of benefit in devising interventions to motivate the uptake of influenza vaccinations.

Furthermore, PF is one of the core concepts in Acceptance and Commitment Therapy (ACT), which is among the third wave of cognitive and behavioral therapies. ACT has been designed to be applicable to a broad range of psychological problems by combining acceptance and mindfulness methods along with activation and behavioral change methods [39]. ACT has been applied successfully in treatments to enhance psychological flexibility, and has led to better health outcomes among individuals with psychoses, anxiety, depression, substance abuse, chronic pain, diabetes, obesity, and epilepsy [39,41,42,43,44]. Indeed, ACT interventions in other populations suggest that ACT is related to an increase in medication compliance among people with psychosis [45] and Human Immunodeficiency Virus (HIV) infections [46]. Thus, ACT might be another reasonable strategy, mediated by fostering psychological flexibility, to use to promote influenza vaccination. However, to our knowledge, there has been no study to date on the relationship between psychological flexibility and influenza vaccination rates, or on the application of ACT on people with CRD. The crucial first step in any such study is to determine differences in the level of psychological flexibility of people with CRD who have been vaccinated and those who have not.

In this study, it is hypothesized that people with low levels of psychological flexibility would allow their desire to avoid uncomfortable emotions, such as anxiety about catching infections from influenza vaccination or fear of needles, to influence their decision to accept an influenza vaccination. On the other hand, people with high levels of psychological flexibility would be more accepting and tolerant of anxiety and would not allow such emotions to influence their decision to receive an influenza vaccination. Accordingly, the study set out to investigate the unique interplay between a person’s level of psychological flexibility and health perceptions in predicting that person’s decision to receive an influenza vaccination.

## 2. Methods

### 2.1. Study Design and Participants

A quantitative, cross-sectional, and correlational survey was employed as the study design in the present research. The target population comprised completely of people with CRD in Hong Kong. The study was carried out in three respiratory medical wards of one public hospital in Hong Kong. Non-probability convenience sampling was used to recruit the sample from the selected settings, where potential participants were accessible to the investigators. A cross-sectional survey was conducted from January to March 2015. The investigators approached patients and invited them to participate in the study. The subject selection criteria were established to maximize the homogeneity of the study sample. Eligible participants were Hong Kong permanent residents aged 18 years or above with a history of chronic respiratory diseases who were able to complete a set of survey questionnaires.

### 2.2. Measures

A self-administered questionnaire with an information sheet was distributed to the participants. The participants completed the questionnaire on the spot. One investigator was available to answer any enquiries about the survey raised by the participants. Most of the participants completed the questionnaire in about 20 min.

A structured questionnaire was used as a research instrument in this study. The questionnaire consisted of three parts. Part one began with eight categorical items assessing the participants’ socio-demographic status (age, gender, marital status, educational attainment, and smoking status), health status (whether they had been diagnosed with other chronic illnesses), and utilization of healthcare services (the number of out-patient clinics visited and hospitalizations in the past 12 months). These items were then followed by a dichotomous item, “vaccinated” and “unvaccinated”, to determine whether the participants had received an influenza vaccination in the preceding 12 months.

Part two consisted of the Acceptance and Action Questionnaire II (AAQ-II) to measure psychological flexibility [47]. The items were rated on a 7-point Likert scale from 1 (never true) to 7 (always true). The scores for items 1, 6, and 10 were reversed to calculate the total score in the 10-item AAQ-II. The scale had no cutoff point. The higher the score, the greater the psychological inflexibility. The internal consistency reliability of the AAQ-II was 0.84, while the 12-month test-retest reliability was 0.79 [47].

Part three was made up of a total of 34 statements from the Champion’s Health Belief Model Scale (CHBMS) [48], organized into five sub-scales measuring perceived susceptibility, perceived severity, perceived benefits, perceived barriers, and cues to action. In the CHBMS, cues to action are defined as reminders, such as mass media communications, interpersonal interactions, or reminder postcards from healthcare providers, to take action to get vaccinated against influenza. The items were measured using a 4-point Likert scale, with 4 being strongly agree and 1 strongly disagree. The higher the total score, the stronger the health perception. The test-retest reliability of the CHBMS ranged from 0.82 to 0.91, while the Cronbach’s coefficient alpha for internal consistency ranged from 0.63 to 0.71 [49].

### 2.3. Statistical Analysis

A statistical analysis was performed using the Statistical Package for the Social Sciences (SPSS) software, version 21.0 (IBM Corp., Armonk, NY, USA). Frequencies and percentages were utilized to illustrate all of the information on the socio-demographics, health status, utilization of healthcare services, and vaccination status of the participants. Because the influenza vaccine refers to an annual vaccination in which people are administered a vaccine that is specific to a given year to protect them against the highly variable influenza virus, an individual is considered to have been vaccinated against influenza if the vaccination occurred within 12 months prior to the individual’s participation in the current study. Mean and standard deviation were used to describe the central tendency and variability of the AAQ-II scores.

In order to achieve the research objectives, the following statistical tests were used with the level of significance (α) specified to be 0.05. First, a chi-square (χ^2^) test for nominal data was conducted to determine differences between vaccinated and unvaccinated sufferers of chronic respiratory diseases with regard to socio-demographic characteristics, morbidity, utilization of healthcare services, and the intention to receive an influenza vaccination in the next influenza season. Second, an independent sample *t*-test for interval data was carried out to compare the mean differences in the AAQ-II scores between the vaccinated and unvaccinated groups. Third, because the independent variables involved various measurement scales, a multivariate logistic regression analysis was used to investigate the predictors of the uptake of the influenza vaccine for people with CRD. Because of the modest size of the sample that was included in the analysis, the variables “Number of visits to an out-patient clinic in the past 12 months” and “Number of hospitalizations in the past 12 months” were transformed into binary covariates instead of being stratified into four categories.

### 2.4. Ethical Considerations

All subjects gave their informed consent for inclusion before they participated in the study. The study was conducted in accordance with the Declaration of Helsinki, and the protocol was approved by the Ethics Committee of the Hong Kong Polytechnic University (Ethics Approval Number: HSEARS20140903001), and the Hong Kong East Cluster Ethics Committee of the Hong Kong Hospital Authority (Ethics Approval Number: HKEC-2014-119). Individual participants received an information sheet and an explanation of the selection criteria, objectives, and procedures of the study. They were assured that participation in the study was voluntary, and that there would be no penalties for choosing not to participate in it. It was specified that no compensation would be offered for participating in this study. The survey was anonymous and participants could withdraw from the study at any time. All of the data that were collected will only be used for the purposes of this study. 

## 3. Results

### 3.1. Comparison of Characteristics between Vaccinated and Unvaccinated Participants

A total of 255 participants completed the questionnaires. Eighty-four of the participants, (32.9%) had received an influenza vaccination and 67.1% (*n* = 171) had not. The data for the two groups of participants are shown in Table 1. The results of the chi-square test for age (χ^2^ = 45.07, degree of freedom (*df*) = 3), smoking status (χ^2^ = 85.38, *df* = 2), the presence of comorbidities (χ^2^ = 46.98, *df* = 2), the number of visits to the Specialist Out-patient Department (SOPD) (χ^2^ = 12.72, *df* = 3), and the number of hospitalizations (χ^2^ = 25.91, *df* = 3) in the preceding 12 months were *p* < 0.05, indicating that the differences in these variables between the vaccinated and non-vaccinated groups were strong and significant.

**Table 1:** Comparison of the characteristics of vaccinated and unvaccinated participants.

Characteristics	Vaccinated Participants *n* = 84 (32.9%)	Unvaccinated Participants *n* = 171 (67.1%)	X^2^ (*df*)	*p*-Value
**Age**	–	–	–	–
18–40	0 (0.0)	36 (21.1)	45.07 (3)	0.000 ***
41–54	15 (17.9)	28 (16.4)	–	–
55–64	18 (21.4)	67 (39.2)	–	–
≥65	51 (60.7)	40 (23.4)	–	–
**Gender**	–	–	–	–
Male	57 (67.9)	117 (68.4)	0.01 (1)	0.928
Female	27 (32.1)	54 (31.6)	–	–
**Marital status**	–	–	–	–
Married	37 (44.0)	76 (44.4)	6.92 (3)	0.074
Non-married	8 (9.3)	34 (19.9)	–	–
Divorced	14 (16.7)	15 (8.8)	–	–
Widowed	25 (29.8)	46 (26.9)	–	–
**Educational attainment**	–	–	–	–
Primary education or below	42 (50.0)	79 (46.2)	3.65 (2)	0.161
Secondary education	32 (38.1)	55 (32.2)	–	–
Tertiary education or above	10 (11.9)	37 (21.6)	–	–
**Smoking status**	–	–	–	–
Currently smoking	5 (6.0)	99 (57.9)	85.38 (2)	0.000 ***
Stopped smoking	24 (28.6)	48 (28.1)	–	–
Never smoked	55 (65.5)	24 (14.0)	–	–
**Other chronic illnesses diagnosed**	–	–	–	–
Yes	59 (70.2)	44 (25.7)	46.98 (2)	0.000 ***
No	25 (29.8)	122 (71.3)	–	–
**Number of visits to an out-patient clinic in the past 12 months**	–	–	–	–
0	8(9.5)	12(7.0)	12.72 (3)	0.005 **
1–2	19(22.6)	33(19.3)	–	–
3–4	26(31.0)	91(53.2)	–	–
≥5	31(36.9)	35(20.5)	–	–
**Number of hospitalizations in the past 12 months**	–	–	–	–
0	13(15.5)	18(10.5)	25.91 (3)	0.000 ***
1–2	28(33.3)	27(15.8)	–	–
3–4	24(28.6)	61(35.7)	–	–
≥5	19(22.6)	65(38.0)	–	–

Note: ** *p* < 0.01; *** *p* < 0.001.

### 3.2. Comparison of the Psychological Flexibility and Health Perceptions of Vaccinated and Unvaccinated Participants

The results of the independent sample *t*-test of the AAQ-II and CHBMS between the two groups are shown in Table 2. Significant differences between the groups were observed in PF (*p =* 0.000) and in some domains of the HBM, including perceived susceptibility (*p* = 0.000), perceived severity (*p* = 0.000), and cues to action (*p* = 0.005). With regard to the HBM variables, the vaccinated participants and non-vaccinated participants did not differ in perceived benefits or barriers to vaccination.

**Table 2:** Comparison of psychological flexibility and health perceptions between the vaccinated and unvaccinated participants.

Measures of Psychological Flexibility/Health Perceptions	Vaccinated Participants *n* = 84 (32.9%)	Unvaccinated Participants *n* = 171 (67.1%)	Statistical and *p* values	
	Mean (SD)	Mean (SD)	*t* (*df*)	*p*-Value
Psychological flexibility ^†^	33.35 (6.01)	45.90 (4.99)	16.06 (253)	0.000 ***
1. It’s okay if I remember something unpleasant.	2.87 (1.08)	4.38 (0.97)	10.53 (253)	0.000 ***
2. My painful experiences and memories make it difficult for me to live a life that I would value.	3.18 (0.95)	4.44 (0.90)	9.94 (253)	0.000 ***
3. I’m afraid of my feelings.	3.33 (0.93)	4.38 (1.07)	7.89 (253)	0.000 ***
4. I worry about not being able to control my worries and feelings.	3.51 (1.06)	4.52 (1.21)	6.72 (253)	0.000 ***
5. My painful memories prevent me from having a fulfilling life.	3.47 (0.88)	4.49 (0.90)	8.48 (253)	0.000 ***
6. I am in control of my life.	3.35 (0.98)	4.79 (1.14)	9.79 (253)	0.000 ***
7. Emotions cause problems in my life.	3.25 (0.95)	4.55 (0.95)	9.98 (253)	0.000 ***
8. It seems like most people are handling their lives better than I am.	3.35 (0.98)	4.68 (1.25)	9.10 (253)	0.000 ***
9. Worries get in the way of my success.	3.56 (0.98)	5.06 (0.95)	11.35 (253)	0.000 ***
10. My thoughts and feelings do not get in the way of how I want to live my life	3.49 (0.91)	4.61 (0.81)	9.28 (253)	0.000 ***
Health Perceptions by CHBMS ^††^	–	–	–	–
Perceived susceptibility ^a^	2.51 (0.37)	2.25 (0.36)	5.12 (253)	0.000 ***
Perceived severity ^b^	2.67 (0.31)	2.32 (0.36)	7.42 (253)	0.000 ***
Perceived benefits ^c^	2.55 (0.60)	2.42 (0.61)	1.52 (253)	0.130
Perceived barriers ^d^	2.13 (0.41)	2.08 (0.42)	0.90 (253)	0.371
Cues to action ^e^	2.48 (0.55)	2.26 (0.58)	2.82 (252)	0.005 **

Notes: ^†^ Psychological flexibility was measured using the AAQ-II, with higher scores reflecting lower psychological flexibility (Experiential avoidance), and the possible score range was: 7–70 [47]. The scores for items 1, 6, and 10 were reversed to calculate the total score in the 10-item AAQ-II. ^††^ Health perceptions were measured by CHBMS ^a,b,c,d,e^, with higher scores reflecting a stronger perception, and the possible score range was: 0–4 [48]. ** *p* < 0.01, *** *p* < 0.001.

### 3.3. Predictors of Influenza Vaccination among People with CRD

The logistic regression analysis showed that age (*p =* 0.000), being a non-smoker (*p =* 0.025), the presence of other chronic illnesses (*p =* 0.001), the number of hospitalizations in the past 12 months (*p =* 0.004), psychological flexibility (*p =* 0.000), perceived susceptibility (*p =* 0.015), and perceived severity (*p =* 0.004) were significant predictors of influenza vaccination. The regression model had a high Nagelkerke R^2^ of 0.707 (see Table 3).

**Table 3:** Predictors of influenza vaccination among people with CRD.

Predictors	OR	95%CI	*p*-Value	Nagelkerke R^2^
Age: ≥65 years	15.20	(4.60, 50.26)	0.000 ***	0.707
Never smoked (*vs.* ever smoking)	4.13	(1.19, 14.33)	0.025 *	
Had one or more chronic illnesses other than COPD	0.16	(0.05, 0.47)	0.001 **	
Number of visits to an out-patient clinic in the past 12 months: ≥3 times	1.40	(0.49, 4.02)	0.532	
Number of hospitalizations in the past 12 months: ≥3 times	1.25	(0.10, 0.63)	0.004 **	
Perceived susceptibility: above mean	3.04	(1.25, 7.42)	0.015 *	
Perceived severity: above mean	3.04	(1.56, 9.81)	0.004 **	
Cues to action: above mean	1.33	(0.56, 3.17)	0.525	
Psychological flexibility: below mean	49.37	(12.18, 200.15)	0.000 ***	

Notes: CI: confidence interval; OR: odds ratio; * *p* < *0.05*, ** *p* < 0.01, *** *p* < 0.001.

## 4. Discussion

In this study, the overall vaccination acceptance rate was 32.9%, which is similar to the rates for people with CRD in the U.S. (33.3%–39.9%) [24,29], but much lower than those for Germany (46.5%) [27], Canada (47.9%) [30], and Spain (63.6%) [25]. Our results revealed that the influenza vaccine uptake rate did not differ according to educational attainment. This finding is inconsistent with the recent U.S. national representative data, which showed that the higher the level of education, the greater the likelihood of vaccination [50]. Although previous studies had found that men and those who indicated that they were single were more likely to be opposed to vaccination [23,29,51], in our study there were no differences in vaccine uptake associated with marital status and gender. This result may be attributable to the fact that the subjects had mostly been recruited from the male wards. Participants who had never smoked or who had stopped smoking tended to be more likely to have received an influenza vaccination compared to current smokers. We also observed that people with other chronic illnesses and those who reported higher frequencies of hospital visits, both SOPD consultations and hospitalizations, were more likely to have been vaccinated against influenza. These findings coincide with the results of earlier studies [20,52,53]. A possible explanation for the finding is that people with poorer health or more severe CRD are more frequently or urgently advised to be vaccinated against influenza, or are more concerned about the consequences of an influenza infection. This notion is incongruent with our finding that the vaccinated individuals perceived themselves to be more susceptible to influenza, and therefore chose to be vaccinated.

With regard to the association between health perception and vaccination status, in the present study vaccinated people with CRD might have perceived that they were a group at risk of getting influenza because of their advanced age and poor lung condition. In addition, the results showed that there was a significant difference between the vaccinated and unvaccinated groups in their overall perception of the severity of influenza. The vaccinated group may have experienced influenza during hospitalization, making them very sick and adversely affecting their usual activities, or have seen others suffering from influenza [54,55]. With regard to the variable of educational attainment, this uniformity may be partly a result of the outbreak of a pandemic of influenza and the prolonged peak seasons for influenza in Hong Kong in recent years [56]. The perceptions of people with CRD regarding their susceptibility to influenza and the severity of influenza are similar to the findings of previous international studies, which found that older people who declined vaccination believed that they were unlikely to catch influenza and that influenza was not dangerous [19].

There were no significant differences between the vaccinated and unvaccinated groups of people with regard to their perception of the benefits and barriers to influenza vaccination. Pointing out the benefits of the influenza vaccine is not enough to persuade unvaccinated people to receive the vaccine, because of their perception of the low risk of getting the flu, and their concern about the side effects caused by the vaccine and the pain associated with an injection [57]. The results showed that cues to action played a comparatively important role in vaccine uptake among people with CRD, which is consistent with the core concept of the HBM that a stimulus must be present in order to trigger health-promoting or disease-preventing behavior [31]. For example, according to a systematic review, vaccination campaigns have had a positive effect on raising rates of influenza vaccination. The review concluded that campaigns with a great variety of components, including education, improved access to vaccines, and the establishment of regulations, were associated with higher vaccination rates [58]. The review further identified that people’s perceptions of their susceptibility to influenza and their perceptions of the severity of influenza were sufficient to prompt them to get vaccinated. Furthermore, previous studies have suggested that health educators should identify strategies to increase the extent to which at-risk individuals understand their health status in order to promote the perception of vaccination as a health behavior [15,17]. However, in the process of building up people’s perceptions of their susceptibility to influenza or of the severity of influenza, one should take care not to create unrealistic perceptions or exaggerated fears about influenza [15]. As a health educator, nurses play a central role in efforts to promote vaccination against influenza. During health talks in promotional activities, nurses could explain the risks and seriousness of catching influenza for people with CRD, the efficacy of the vaccine, and the possible side effects of the influenza vaccine.

The PF of the unvaccinated individuals in this study was found to be 45.9, which is comparable to previous findings for people with anxiety disorder (38.14) [59], but lower than for people with obesity (55.1) [60], and higher than for cancer patients (20.19) [61]. Unvaccinated participants had significantly lower levels of psychological flexibility than participants who had received an influenza vaccination in the past 12 months. In theory, unvaccinated individuals would be motivated to avoid or reduce uncomfortable internal experiences. In other words, such people will choose the path of least resistance in order to minimize their experience of discomfort, which means refusing to be vaccinated against influenza. Previous research supports this line of thought, with the low use of adaptive coping or non-committed actions being associated with more psychopathology via differences in psychological inflexibility as a general characteristic [62].

The regression analysis suggested that the variables of being over 65 years of age, a non-smoker, the presence of comorbidity, the perception of oneself as being more susceptible to influenza than others, the perception of influenza as a severe infection, and higher psychological flexibility were predictors of the acceptance of influenza vaccination. A high level of perceived susceptibility and severity may indicate that people felt anxious about getting infected with the influenza virus, as they perceived themselves to be more susceptible to infections than others. This may have motivated them to avoid the kinds of uncomfortable experiences that they had had in the past, such as frequent hospitalizations and severe infections. Prior research has pointed out that a perceived threat (high susceptibility and/or high severity) is associated with more negative emotions [63]. It is possible that in an attempt to resolve this conflict and reduce the experience of negative emotions, psychologically flexible people with CRD were motivated to commit to action (acceptance of influenza vaccination) in order to pursue their valued ends (securing protection from influenza viral infections). It should be noted that the strength of predictors cannot readily be compared, as the possible range of values of the measurement scales differed.

As shown in the results of this study, despite the implementation of a multifaceted, evidence-based, locally designed promotion program in Hong Kong, the knowledge and beliefs of psychologically inflexible individuals about the influenza vaccine did not change. A recent study also described similar findings and suggested that providing information concerning influenza vaccination may have little impact on strongly held beliefs, even when the information is highly customized [64]. In this regard, it is worth exploring new strategies to motivate those who persist in refusing the influenza vaccine to accept vaccination. This study identifies a new variable in the field of influenza vaccination research—PF, a component of ACT that has not previously been investigated. The data from this study suggested that the degree of PF contributes to the decision of people with CRD to accept or refuse to be vaccinated against influenza. As stated in these studies, a diminished level of PF can potentially explain why people of strong convictions are so resistant to changing their beliefs and accepting disease prevention interventions. The research evidence suggests that PF is effectively increased through ACT and that in these interventions positive treatment outcomes are often mediated by improved PF [39,41,44].

Vaccination programs are becoming a public health priority. The findings have important implications for both policy and practice. Social and psychological factors significantly influence influenza vaccination uptake rates; therefore, the inclusion of psychological aspects in vaccine promotion programs is critical to improving vaccination coverage. We suggest that future research could explore whether the prevalence of influenza vaccination among people with CRD could be increased by enhancing psychological flexibility, such as through using acceptance and commitment therapy.

ACT is not only an approach that can be applied at the level of therapy for human cognition, but is also a model of psychopathology that is rooted in pragmatic philosophy (functional contextualism) and theory (relational frame theory) [39]. This model proposes that at the core of most behavioral problems is experiential avoidance (EA), which is the opposite orientation of PF. One of the key processes of EA is a refusal to accept or an unwillingness to experience negative thoughts, emotions, or sensations. EA is thought to be associated with suffering, as attempts from patients to control, change, or eliminate internal experiences that are not under their behavioral control can lead the patient away from behaviors that are more consistent with their values. In the case of the acceptance or refusal of influenza vaccination by people with CRD, individuals with lower levels of PF were more reluctant to accept the influenza vaccine. They also reported higher levels of concern about the side effects of the influenza vaccine and about suffering from catching influenza from the vaccination, and a greater fear of needles. Previous studies also support the association of EA with poorer adherence to treatment or health promotion activities [41,42,43].

As a model of therapy, the main goal of ACT is to increase psychological flexibility as opposed to eliminating negative experiences. The positive techniques put forward in ACT, such as acceptance and cognitive defusion, help individuals come into fuller contact with their negative private experiences while behaving in a way that serves their values, therefore attaining a positive outcome [39]. As mentioned at the outset, ACT interventions were associated with higher medication compliance rates in people with psychosis and HIV infections [45,46]. In these studies, patients were taught to identify and abandon internally oriented strategies, to accept the presence of difficult thoughts or feelings, to learn to just notice the occurrence of these private experiences without struggling with them, arguing with them, or taking them to be literally true, and to focus on overt behaviors that produce valued outcomes, which is adherence to the treatment regime. In fact, people with psychosis and HIV infections share something in common with people with CRD: Their disease is of a chronic nature and is associated with adverse physical, psychological, and emotional experiences, and sufferers have a tendency to avoid certain behaviors. The mediational components of these studies also support the hypothesis that acceptance of the influenza vaccine is promoted via an increase in psychological flexibility. Therefore, similar ACT approaches might be applicable to people with CRD, mediated by enhancing their PF, to foster their acceptance of the influenza vaccine. In the future, clinicians might consider incorporating ACT into routine pulmonary rehabilitation programs (PRP). PRP allow more therapeutic contact time between people with CRD and clinicians, making it feasible to use ACT to promote influenza vaccination.

The present study has several limitations, and caution must be exercised in generalizing its results. This is because the sample was a convenience sample and was not diverse; moreover, it was obtained from only one local hospital. The use of self-reporting in this study might have resulted in recall bias. In addition, unvaccinated people might have been less willing than vaccinated people to participate in such a research study, as has been reported in previous studies. Both of these factors might have contributed to an overestimation of vaccination in our study. Furthermore, although most of the factors influencing individuals to get vaccinated against influenza were covered, some important ones, such as previous vaccination status, were not captured in the current study. Indeed, as documented in a prior study, previous vaccination status was associated with a future intention to receive influenza vaccination [65].

## 5. Conclusions

In conclusion, to our knowledge, this is the first study to take factors of two different models, the HBM and PF, to make comparisons between vaccinated and non-vaccinated people with chronic respiratory diseases. The overall prevalence of influenza vaccination in our sample was 32.9%. Our study showed that among people with CRD, smoking status, hospitalization, perceived susceptibility, and psychological flexibility were critical determinants of the likelihood of getting vaccinated against influenza. For the first time, psychological flexibility among people with CRD was identified as a significant contributing factor in influenza vaccination. Acceptance and Commitment Therapy may be helpful in motivating patients to receive the vaccination.
